# Hyperacute thrombolysis with recombinant tissue plasminogen activator of acute ischemic stroke: Feasibility and effectivity from an Indian perspective

**DOI:** 10.4103/0972-2327.44556

**Published:** 2008

**Authors:** S. R. Sharma, Nalini Sharma

**Affiliations:** Department of Neurology, S. R. M. S. Institute of Medical Sciences, Utter Pradesh, India

**Keywords:** r-tpa, stroke, thrombolysis

## Abstract

Given the constraints of resources, thrombolysis for acute ischemic stroke (AIS) is under evaluation in developing countries like India, especially in areas such as western Utter Pradesh, where it is overly crowded and there is poor affordability.

**Aim:**

This study was done to evaluate recombinant tissue plasminogen activator r-tpa in acute ischemic stroke in hyper acute phase, in selected patients of western Utter Pradesh, in terms of feasibility and effectivity.

**Design:**

Open, non randomized study.

**Materials and Methods:**

Thirty two patients were classified using Trial of ORG 10172 in Acute Stroke treatment (TOAST) criteria (large artery atherosclerotic = 8; cardio embolic = 6; small vessel occlusion = 14; other determined etiology = 2; undetermined etiology = 2). The mean time to reach the hospital was 2 h (1.15-3.0), the mean door to CT scan 20 min (10-40) and door to r-tpa injection was 30 min (24-68). The National Institute of Health Stroke Scale (NIHSS) scores ranged from 11-22 (mean 15.5 +2.7). The dose of r-tpa administered was 0.9 mg/kg.

**Results:**

Twenty one patients (65.6%) showed significant improvement on the NIHSS score, at 48 h (4 points). (Mean change = 10; range = 4-17). At one month, 25 (78%) recorded improvement on the Barthel index (mean change = 45%). One developed frontal lobe hemorrhage and another developed recurrent stroke; one died of aspiration; and four showed no improvement. Modified Rankin score (m RS) was administered at the end of three months to 28 patients (90%); however, the rest could not be directly observed. The average modified Rankin Score was 1.2 (0-2).

**Conclusions:**

Hyperacute thrombolysis was found feasible and effective in selected patients with AIS from western Utter Pradesh and who had poor affordability.

## Introduction

Acute ischemic stroke (AIS) is an area in neurological clinical practice, which has probably seen the greatest strides as far as an understanding of pathogenesis is concerned. This has led to advancement in the treatment options available for patients suffering from ischemic stroke. Plenty of nihilism that surrounded strokes is gradually being lifted along with the ignorance related to the condition, from both the minds of patients and the treating physicians. Most therapeutic options for AIS have one common feature; they generally have to be administered within a very narrow and specific time window. This is extremely pertinent, if the drug related adverse effects have to be minimized and therapeutic benefits maximized.

A study done by the National Institute of Neurological Disorders and Stroke (NINDS) on the intravenous use of recombinant tissue plasminogen activator (r-tpa), within three hours of AIS, has shown the benefits of this form of treatment.[1] In order to be effective, the protocol for institution of r-tpa therapy must be strictly followed. The level of this form of therapy is level I.[2] When all accumulated evidence for r-tpa therapy is considered, the relative risk reduction by the therapy is 44%, absolute risk reduction is 13% and the number needed to treat to save one person from death or disability is seven.[3]

Many countries now routinely thrombolyse all patients of AIS, who present within the three-hour window period and do not have any contraindication. However, many stroke units, especially those in the developing nations like India, especially in western Utter Pradesh, are hampered by constraints of resources and lack of awareness and affordability. The feasibility of thrombolysis is still being evaluated at these centers, as is being done in our study. Hyperacute thrombolysis for stroke was started in the year 1995-96 in India and in March 2002 at the All Institute of Medical Sciences (AIIMS).[4]

Our study was slightly delayed by one to two years. We assessed the outcomes of the patients treated with IV r-tpa, including Intra Cerebral Hemorrhage (ICH) and other events, especially affordability in the population of western Uttar Pradesh. The overall purpose of this study is to report the current clinical practice of IV r-tpa for AIS in North India, especially western Utter Pradesh.

## Materials and Methods

We present the detailed data of the 32 patients treated at SRMS Institute of Medical Sciences (SRMSIMS), in whom an adequate follow-up of six months is available. All the patients were studied prospectively.

The variables that were specifically recorded for each patient treated with r-tpa included time of onset of symptoms, time of arrival at the emergency department, time of CT scan examination and the time of r-tpa administration. An extensive neurological examination, including the baseline NIH stroke scale (NIHSS) was performed in all the patients. Other parameters noted were the demographic profile, stroke risk factors, baseline CT scan findings and blood pressure measurements [Table 1].

**Table 1:** Patients' characteristics

Age	66 (38-78)
Sex	18 : 14
Hypertension	20 (62.5%)
Diabetes mellitus	13 (40.6 %)
Hypercholesterolemia	5 (15.6%)
Atrial fibrillation	7 (21.9%)
Congestive heart failure	5 (15.6%)
Coronary artery disease	4 (12.5%)
Prior stroke	6 (18.6%)
Smoking	10 (31.2%)
Mean admission blood pressure	160/90
Mean maximum pretreatment blood pressure	170/94
NIHSS	15.5 ± 2.7(11-22)

Potential benefits and significant risks, specifically the fourfold greater risk of symptomatic intracerebral hemorrhage[5] after thrombolytic treatment were discussed with all the patients and/or their families and informed consent was obtained. The r-tpa protocol was based on the protocol published by the American Heart Association and the American Academy of Neurology.[6,7] Inclusion and exclusion criteria are shown in Table 2. All patients had pretreatment CT scans that were read by the attending neurologist together with the radiologist. Each of our patients received 0.9 mg/kg of intravenous r-tpa, up to a maximum of 90 mg, based on the estimated or actual body weight. Ten per cent of this calculated dose was injected as a bolus and the remainder infused over an hour. Heparin and aspirin were withheld for the first 24 h after r-tpa administration in all our patients. Hypertension was treated using intravenous labetolol or intravenous enalapril. Occasionally, injection nitroglycerine or nitroprusside was required.

**Table 2:** Criteria for the use of tissue plasminogen activator r-tPA in the treatment of acute ischemic stroke

Clinical inclusion criteria
Age >18 years
Consent by patients or surrogate
Onset of symptoms to time of drug administration <3 h
Measurable neurological deficit defined as impairment of language, motor function, cognition and/gaze, vision or neglect
Score for stroke severity >4 on the National Health Stroke Scale
**Exclusion criteria**
Sustained Blood Pressure (BP) >185/110 mmHg
Platelets <100,000; HCT 25%, glucose <50 or >400.
Use of heparin within 48 h and prolonged PTT, or elevated INR
Minor stroke symptoms (NIHSS score <4)
Rapidly improving symptoms
Prior stroke or head injury within three months; prior intracranial hemorrhage
Recent myocardial infarction
Gastrointestinal bleeding in the preceding 21 days
Major surgery in the preceding 14 days
Coma or severe obtundation with fixed eye deviation and complete hemiplegia or NIHSS score >22
Computed tomography (CT) exclusion criteria
CT scan showing no hemorrhage or significant edema

PTT - Partial Thromboplastin Time, INR- International Normalised Ratio

The results of follow-up CT scans or MRI scans were recorded in all the patients thrombolysed. The use of aspirin, clopidogrel, heparin, actirom and antihypertensive during the hospital stay was noted (see table 3). The peak blood pressure during r-tpa administration as well as during the first 24^th^ hour after the infusion was recorded. Using TOAST criteria, stroke subtype was determined by the following classification: 1) Large artery atherosclerosis 2) Cardio-embolic 3) Small vessels occlusion 4) Stroke of other determined etiology and 5) Stroke of undermined etiology. We obtained telephone or clinic follow-up with the patient and the caregiver in all the 32 cases and assessed the Barthel Activities of Daily Living Index. For analysis, we defined good outcome as an improved NIHSS of >4 and a Barthel Index of >75%. At three months, 25 patients (78%) showed improvement on the Barthel index (mean change = 55%), one developed symptomatic ICH improved on conservative management. One patient died due to fatal ICH, with aspiration pneumonia. One of the three patients who had hemorrhagic conversion improved but the other two did not improve.

**Table 3:** Clinical characteristics of the study population

Received antihypertensive treatment	26 (81.2)
Received anticoagulation	6 (18.7)
Received anti platelets	32 (100)
Received transfusion	-
Length of hospitalization	11+_9 days
Infarct of follow-up CT/MRI	32 (100)
Hemorrhagic conversion	3 (9.4)
Symptomatic intracerebral hemorrhage	1 (3.1)
Fatal intracerebral hemorrhage	1 (3.1)
Mean length of follow-up	8.5 ± 3 months

Figures in parentheses are in percentage

## Results

Thirty two patients received IV r-tpa for AIS between September 2004 and July 2007. We observed 584 patients suspected of having an acute ischemic stroke during this period. The most common reasons for disqualification from thrombolytic therapy were as follows: exceeding the window period 48%, ICH 20%, minor or rapidly resolving symptoms (10%), and non stroke diagnosis (10%). Of the patients who could qualify to receive thrombolysis, 40% could not be thrombolysed, since they could not afford r-tpa. Affordability is a major drawback in the use of r-tpa.

The mean age of our patients was 66 years, ranging from 38 to 78. Out of the 32 patients who were thrombolysed, 18 were males. Comorbid illnesses in the form of hypertension, diabetes and hypercholesterolemia were present in 20 (62.5%), 13 (40.6%), and five (15.6%) respectively. Additionally, atrial fibrillation was present in seven (21.9%), congestive heart failure in five (15.6%) and coronary artery disease in four (12.5%). There was history of prior stroke in six (18.6%). The mean blood pressure at admission was 160/90 mmHg and the mean pretreatment peak blood pressure was 170/94. Ten patients (31.2%) were smokers.

The mean baseline NIHSS was 14 ± 2 (range 8-22). Using TOAST criteria, the patients were classified into: large artery atherosclerotic - eight; cardio emboli - six; small vessel occlusion - 14; other determined etiology - 2; undetermined etiology - 2. The mean time to reach emergency was 2 h (1.15-3). The mean door to rtPA injection time was 30 min (24-68). The mean door to CT scan time was 20 min (10-40 min). The NIHSS scores ranged from 11 to 22 min (mean 15.5 ± 2). The PT, aPTT and platelets count were obtained prior to thrombolysis. Early signs of infarction on CT scan were seen in 15 of the 32 (46.8%) patients. There was sulcal effacement in seven, insular ribbon sign in three and loss of gray white matter differentiation in five. An infarct in the follow-up CT or MRI was present in all the patients. The mean length of hospitalization in our patients was 11 days. During hospital stay, antihypertensive had to be given to 26 (81.2%) patients. Anti platelet drugs were given to all 32 patients, while six (18.7%) received anticoagulants. Blood transfusion was required in none of the cases. Hemorrhagic conversion was noted in three (9.4%) symptomatic intracranial hemorrhage or fatal hemorrhage occurred one each in our study.

Twenty one patients (65.6%) recorded significant improvement on NIHSS at 48 h (>4 points) (mean change = 10, range = 4-17). At one month, 25 (78.0%) improved on Barthel index (mean change=45%). Of these, 11 (44%) of the 25 patients improved; 35% of total number (32) achieved 95% scores on Barthel index, indicating near normal functional status. Of the stroke subtypes, cardioembolic and small vessel occlusions did better than others. Of the four patients who showed no improvement, two were lacunars strokes. One patient developed frontal lobe hemorrhage and recurrent stroke; one died of aspiration and four showed no improvement. The mean length of follow-up after discharge was 8.5 ± 3 months. Modified Rankin score (m RS) was administered at the end of three months to 28 patients (90%); the rest could not be directly observed. The average modified Rankin Score was 1.2 (0-2). One patient developed pneumonia and recovered with therapy.

## Discussion

Despite the demonstration of benefits from thrombolytic treatment with intravenous r-tpa by the NINDS trial as early as in 1995, this form of therapy has been underused in developing countries like India and even in overly populated states such as Utter Pradesh. The reluctance to aggressively thrombolyse patients of acute ischemic stroke has been attributed to several factors including delay in arrival of patients to emergency, limited resources, ignorance or disbelief amongst neurologists with regard to the efficacy of this mode of treatment and fear of serious r-tpa-related complications like hemorrhage. We succeeded in treating 32 patients of acute ischemic stroke with IV r-tpa, over a period of three years. Importantly, we were unable to thrombolyse 48% of otherwise eligible patients, due to delay in their arrival. Several studies have been tried to analyze the causes of such delays, but they been conducted in developed countries which are very different from India.[8–13]

The mean age of patients in our study was 66 years, whereas the mean age of patients in the NINDS trial was 67 years. Advanced age increases the odds of a poor outcome after thrombolysis.[14–15] However *post hoc* analysis of the NINDS data showed r-tpa to be beneficial for patients in all strata of age. As the age of incidence of stroke increases, it becomes important to decide if older patients should be denied this potentially disability-avoiding therapy.

Our patients had a mean baseline NIHSS of 14, which is similar to that of patients in the NINDS r-tpa trial. The stroke sub types by final diagnosis was most often (35% patients) in small vessel disease. The NINDS trial and a few subsequent studies have shown that IV r-tpa within the three hours window is similar between different stroke subtypes. It has been recommended that extensive diagnostic evaluation to determine stroke subtypes before thrombolysis is neither required nor justified.

Patients arrived in the emergency department at an average of 120 min after symptom onset. A CT scan was performed within 20 minutes and IV r-tpa was administered within an average of 30 min from the time of arrival at the emergency department. A significant difference between our study and the NINDS trial is that half of the randomized patients in the NINDS trial were treated within 90 min of stroke onset. However, the response to the treatment observed in the NINDS trial between patients treated under 90 min and between those treated between 90 and 180 min did not differ future direction for widening the window period. This underlies the importance of not withholding treatment from patients, even if they present beyond 90 min of the onset of symptoms. We were able to treat patients within an average of 30 min from the time of arrival at the emergency department. This is contrary to the popular perception that it is generally not possible to treat patients within the time window of 180 min in busy and constrained hospitals of India, like in our study, unless they arrive early. Recent NIH consensus guidelines recommend a “door to needle” time of 60 min or less for acute stroke patients.

An improvement of four points or more on the NIHSS at 48 h was seen in 65.6% of our patients, while a Barthel Index of 75 or more at one month was present in 78% patients. It is important to note here that long term benefits as observed from the Barthel Index is significantly more impressive than the immediate benefits as reflected from the 48 h NIHSS. The reluctance of many neurologists to treat patients is based on this premise of insufficient immediate improvement in outcome. However, it is equally important not to lose sight of the delayed benefits, especially considering the tremendous impact that stroke morbidity has on the individual, caregivers and society as a whole.

The preliminary data from India, with reference to western Utter Pradesh, shows that hyperacute thrombolysis in acute ischemic stroke is both feasible and effective but still not affordable to a large segment of the population, due to poverty and lack of awareness of its importance and usefulness. It also emphasizes the importance of increasing awareness about stroke and its acute management. More significantly, until patients start presenting more promptly for treatment, it needs to be understood that thrombolysis can still be provided in hospitals if a dedicated team of neurologists endeavors to reduce the “door to needle” time to the minimum possible.

The limitation of the present study is the lack of control group, which will impede the assessment of the efficacy of thrombolytic. However, since the study was conducted to essentially evaluate the feasibility and effectivity in western Utter Pradesh (India) which is a poverty stricken pocket and hence affordability of r-tpa is a major issue, the limitation is the sample size. We accede that it is a small number to draw any definite conclusions. However, the observation does reveal that even in this small number of patients, hyperacute thrombolysis can be undertaken in carefully screened patients of AIS. A future large trial needs to be undertaken with a blinded, controlled and randomized design, to substantiate our findings.
